# Sustainability in Youth: Environmental Considerations in Adolescence and Their Relationship to Pro-environmental Behavior

**DOI:** 10.3389/fpsyg.2020.582920

**Published:** 2020-11-02

**Authors:** Audra Balundė, Goda Perlaviciute, Inga Truskauskaitė-Kunevičienė

**Affiliations:** ^1^Environmental Psychology Research Centre, Institute of Psychology, Mykolas Romeris University, Vilnius, Lithuania; ^2^Department of Psychology, Faculty of Behavioural and Social Sciences, University of Groningen, Groningen, Netherlands

**Keywords:** biospheric values, environmental self-identity, personal norms, environmental behavior, environmental considerations, adolescents

## Abstract

Adolescents today face the negative outcomes of climate change, and their pro-environmental behavior is crucial to mitigate these negative outcomes. Yet, we know little about what influences adolescents’ pro-environmental behavior. Research shows that people’s biospheric values and environmental self-identity, elicit personal norms to act environmentally friendly, which can induce a wide range of pro-environmental actions. Yet there is no evidence that these factors can influence pro-environmental behavior of adolescents, because this has only been studied for adults. Given that in adolescence, values, identities and moral structures undergo intense development, the question is whether these factors can motivate adolescents to act pro-environmentally. To address this question, we have conducted three studies with adolescents in Lithuania (Study 1: *N* = 256; Study 2: *N* = 349; Study 3: *N* = 905). We found support that adolescents’ biospheric values and environmental self-identity were associated, via personal norms, with a wide range of pro-environmental behaviors, including recycling, environmentally friendly traveling, purchasing environmentally friendly goods and drinking tap water. Based on theory and the current findings, we suggest directions for policies aimed at promoting pro-environmental behavior of adolescents.

## Introduction

Like no other generation, the youth today are exposed to grand environmental challenges (Faustini, 2014). Adolescents worldwide are rising and initiating social movements to urge policy makers to tackle environmental challenges such as climate change (e.g., Fridays for Future). This may signal that today’s youth are concerned about the anthropogenic climate change and hold moral standards that motivate them to act pro-environmentally. But is this indeed the case?

**FIGURE 1:**
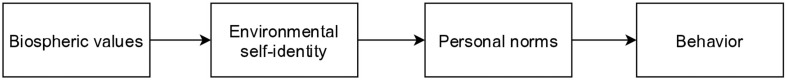
The relationship between environmental considerations and behavior (adapted from van der Werff and Steg, 2016).

The Value-Identity-Personal norm theoretical model suggests that people’s general environmental considerations such as biospheric values strengthen environmental self-identity and elicit moral obligation to act environmentally friendly (Figure 1; Ruepert et al., 2016; van der Werff and Steg, 2016). Yet, this relationship has only been tested for adults and never for adolescents. Adolescents’ values, identity and moral structures undergo intense development and are not stable yet (Moshman, 1999; Wigfield et al., 2006). This raises a question to what extent, if at all, adolescents hold personal norms to act pro-environmentally that are rooted in their biospheric values and environmental self-identity. Such knowledge is needed to develop evidence-based age-tailored policies to foster adolescents’ pro-environmental behavior (United Nations General Assembly [UNGA], 2018).

Pro-environmental behavior is aimed at protecting the environment or at least not harming it (Lange and Dewitte, 2019). Values are people’s general goals or ideals in life that transcend situations and guide behavior (Schwartz, 1977, 1992). Four values have been found to be important to explain pro-environmental behavior, namely biospheric (caring for nature and the environment), altruistic (caring for other people), egoistic (caring for personal resources), and hedonic values (seeking pleasure and comfort; de Groot and Steg, 2007a,b; de Groot et al., 2012; Steg et al., 2014). Biospheric and altruistic values are part of self-transcendence values (i.e., concern for the wellbeing of others), and egoistic and hedonic values are part of self-enhancement values (i.e., concern for personal interests and welfare; Schwartz, 2012a).

Particularly people’s strong biospheric values have been found to be important to explain multiple pro-environmental behaviors (de Groot and Steg, 2007a,b; Steg et al., 2012, 2014; Merrill et al., 2018). Studies found positive relationships between biospheric values and recycling and environmental activism (Balundë et al., 2019), energy conservation (de Groot et al., 2012; Sahin, 2013) and acceptability of policies to reduce car use (Hiratsuka et al., 2018; Ünal et al., 2019).

Because biospheric values reflect very general goals in life, they are related to behaviors mostly indirectly via intermediate factors, in particular environmental self-identity (van der Werff et al., 2014a). Environmental self-identity is the extent to which a person sees her/himself as someone who acts environmentally friendly (van der Werff et al., 2013b). The more people endorse biospheric values, the stronger is their environmental self-identity (van der Werff et al., 2014b). Together biospheric values and environmental self-identity can elicit people’s personal norms to act pro-environmentally (van der Werff et al., 2013a,b). Personal norms are internalized moral standards (Olkinuora, 1972; Schwartz, 1977), expressed as a sense of moral obligation to protect the environment (Steg et al., 2011). Personal norms to protect the environment are related to various pro-environmental actions, such as intentions to use green energy, preferences for sustainable products and willingness to reduce car use (Nordlund and Garvill, 2003; van der Werff et al., 2013a; Barbarossa and De Pelsmacker, 2016).

In sum, research suggests that people may hold moral obligations to act pro-environmentally, which are rooted in their biospheric values and environmental self-identity. These moral obligations in turn guide people’s pro-environmental behavior (Ruepert et al., 2016; van der Werff and Steg, 2016; Figure 1). The full chain of relationships between biospheric values, environmental self-identity and personal norms has been demonstrated for pro-environmental behavior at work (Ruepert et al., 2016), participation in renewable energy projects (van der Werff and Steg, 2016) and tourists’ pro-environmental behavior (Xu et al., 2019).

However, these relationships have only been tested for adults so far, but never for adolescents. This is an important gap in the literature, because there is an urgent need for the youth of today to engage in many different sustainable behaviors. Therefore, it is critical to study general antecedents that influence adolescents’ environmental behaviors, to effectively address the environmental crisis. Noteworthy, biospheric values and environmental self-identity in adolescence could potentially be fostered, for example, by means of environmental education (Caduto, 1983, 1985). Yet, to estimate whether such policies could be effective in promoting adolescents’ pro-environmental behavior, it is crucial to study adolescents’ biospheric values and environmental self-identity and their relationships with personal norms and the actual pro-environmental behavior.

Interestingly, pro-environmental behavior tends to decline from childhood to adolescence (Evans et al., 2007; Krettenauer, 2017; Wray-Lake et al., 2017; Krettenauer et al., 2019; Otto et al., 2019) and again increase in adulthood (Grønhøj and Thøgersen, 2009; Otto and Kaiser, 2014). Similarly, adolescents tend to see pro-environmental behavior as less obligatory then their younger counterparts (Krettenauer, 2017). This suggests that personal norms to act pro-environmentally and eventually pro-environmental behavior are not yet stable in adolescence. There is initial evidence that adolescents’ pro-environmental behavior is related to their personal norms to act pro-environmentally (Matthies et al., 2012; Uitto et al., 2015; Collado et al., 2017). Yet it has not been studied whether these norms and behaviors are rooted in adolescents’ biospheric values and environmental self-identity.

Studies in various cultures have shown that universalism values, which encompass caring for nature and the environment as well as other people (Schwartz, 2012a), can already be detected in adolescents and distinguished from their other values (Schwartz et al., 2001, Schwartz, 2012b; Liem et al., 2010; Cieciuch et al., 2013; Paez and De-Juanas, 2015). At the same time, these values seem to be less prioritized in adolescence than later in life and weaker than other values, in particular self-enhancement values (Schwartz, 2012b; Vecchione et al., 2019). This suggests that biospheric values in particular may still be developing in adolescence. Furthermore, adolescents’ identity structures are not stable yet as adolescents are still exploring their identities, including through social interactions with parents and peers (Erikson, 1968; Crocetti et al., 2008; Klimstra et al., 2010; Meeus et al., 2010; Luyckx et al., 2011; Crocetti, 2017; Kaplan and Garner, 2017). Given that values, self-identity and moral standards of adolescents are still changing, we designed the study to test these key constructs in adolescence and their relationships with adolescents’ pro-environmental behavior.

Importantly, acting sustainably requires a large variety of actions, from recycling to supporting environmental policy. Research suggests that specific constructs such as behavior-specific self-identity (e.g., “I see myself as someone who recycles) and personal norms (e.g., “I feel morally obliged to recycle”) can predict the respective behavior (i.e., recycling; Geiger et al., 2019). Yet, it can be not very practical to look at only specific constructs, because of the wide range of behaviors that need to be promoted to address climate change. Values, on the other hand, are general constructs that could potentially predict a large variety of behaviors (Dietz, 2015). There is some initial evidence that environmental values influence many pro-environmental behaviors of adolescents, including cycling to school and other environmentally friendly everyday activities (Ojala, 2013). However, only the aggregate of the different behaviors was examined in this study, and not the effects of values on each individual behavior. Based on the compatibility principle (Ajzen, 1996; Trope and Liberman, 2010), one could argue that because values are measured on a general level, they only influence general categories of behaviors, but not concrete behaviors. To rule out this alternative explanation, we will test to what extent general environmental considerations, namely biospheric values and environmental self-identity, drive, via personal norms, various pro-environmental behaviors. We test this for behaviors at different levels of specificity – from general categories of behaviors to very specific actions.

The current research was designed to test the relationships between environmental self-identity, personal norms and pro-environmental behaviors in adolescence. We tested these relationships across three studies that targeted different pro-environmental behaviors, from general to specific. Specifically, we tested the relationships between adolescents’ environmental considerations and more general behaviors such as recycling waste, choosing environmentally friendly transportation means and purchasing sustainably produced products (Study 1) and more specific behaviors, such as recycling non-refundable plastics, cycling to school, and purchasing organic food products (Study 2) and drinking tap instead of bottled water (Study 3). In all three studies, we first tested whether biospheric values can be distinguished from other values (i.e., altruistic, egoistic and hedonic); and whether biospheric values, environmental self-identity and personal norms can be empirically distinguished from each other in adolescence sample. Then, we tested the extent to which biospheric values, environmental self-identity and personal norms can explain the different types of pro-environmental behaviors of adolescents.

If environmental considerations indeed guide general as well as specific pro-environmental behavior in adolescence, interventions could focus on, for example, strengthening biospheric values (e.g., through parents’ and teachers’ role modeling; environmental education). Adolescence could be an especially good time for such interventions, because environmental considerations in adolescence are still developing and may be more susceptible to change than in adulthood. Consequently, adolescents will have a stronger intrinsic motivation to act pro-environmentally and there may be less, need for interventions based on financial (dis) incentives, laws and regulations. Also, adolescents’ environmental considerations could be a gateway to many actions needed to combat climate change, such as changing traveling behavior and consumption habits, reducing energy use at home or at school, and recycling, among others (Intergovernmental Panel on Climate Change [IPCC], 2018).

## Materials and Methods

### Participants and Procedure

**FIGURE 2:**
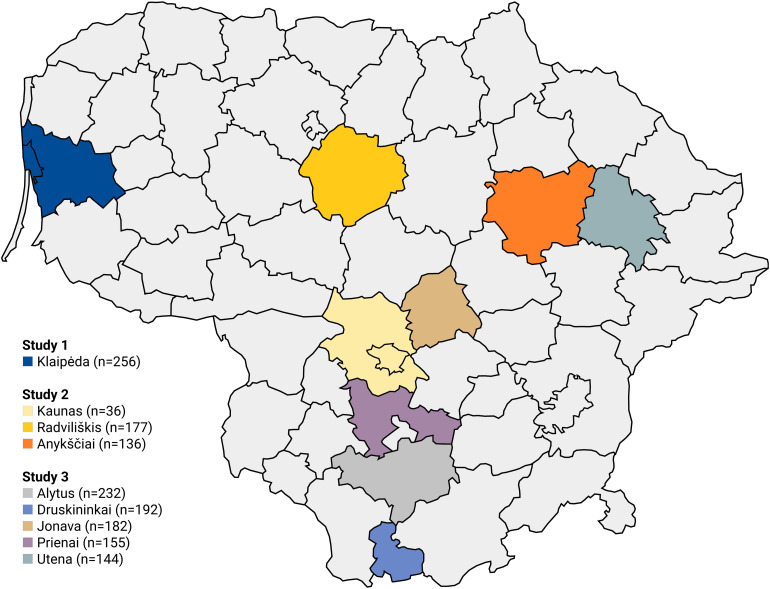
Geographical representation of regions where three studies were conducted.

The three studies were conducted in nine different municipalities of Lithuania covering different geographical regions, including urban and rural areas (Figure 2), enabling us to test the robustness of the findings. Participants completed online questionnaires at school, in computer labs, during a pre-scheduled meeting, using either desktops at school (Study 1) or tablets provided by the research team (Study 2 and 3). The survey time was strictly limited to avoid interference with participants’ activities, such as classes or recess time.

Participants reported demographic characteristics (see Table 1), followed, in a random order, by measures of values, environmental self-identity, personal norms to act pro-environmentally and pro-environmental behavior. All studies were part of a larger research project on adolescents’ pro-environmental behavior. We only discuss the measures that were included for the purposes of the current studies.

**TABLE 1:** Summary of sample characteristics.

**Study**	**Year**	**Number of schools**	**Removed cases due to missing data**	**Refused to participate**	**Response rate**	**Age**	**Gender**	
							**Boys**	**Girls**
Study 1 (*n* = 256)	2016	5	–	2	99.22%	14–18 (*M* = 15.33, SD = 0.91)	116 (45.3%)	140 (54.7%)
Study 2 (*n* = 349)	2018	3	6	1	99.22%	13–18 (*M* = 16.07, SD = 0.99)	158 (45.3%)	191 (54.7%)
Study 3 (*n* = 905)	2019	5	26	5	99.47%	13–17 (*M* = 15.23, SD = 0.68)	414 (45.7%)	491 (54.3%)

### Measures

Participants’ values were measured with a short version of the Schwartz’s values instrument (Schwartz, 1992; de Groot and Steg, 2007a; Steg et al., 2012). Participants indicated on a nine-point scale to what extent different values are important guiding principles in their lives, from *-1 opposed to my guiding principles*, *0 not important*, to *7 of supreme importance*. Biospheric values scale consisted of four items, e.g., “Unity with nature: fitting into nature.” We took the mean of these items to calculate biospheric values; higher values indicate stronger biospheric values (Study 1, α = 0.89, *M* = 4.42, SD = 1.84; Study 2, α = 0.87, *M* = 4.35, SD = 1.86; Study 3, α = 0.88, *M* = 5.12, SD = 1.70). Confirmatory factor analyses in all three studies showed that biospheric values can be empirically distinguished from other values, namely altruistic, egoistic and hedonic values (Supplementary Material 1). Specifically, the items measuring biospheric values correlated stronger with the biospheric-values scale than with the other value scales, after controlling for self-correlations^1^.

We used an established measure of environmental self-identity, which consists of three items (e.g., “I see myself as an environmentally friendly person”; van der Werff et al., 2013a,b). Participants indicated to what extent they consider themselves as a person who acts pro-environmentally, from *1 – totally disagree* to *5 – totally agree.* We took the mean of these items to calculate environmental self-identity; higher values indicate stronger environmental self-identity (Study 1, α = 0.78, *M* = 3.33, SD = 0.84; Study 2, α = 0.73; *M* = 3.60, SD = 0.64; Study 3, α = 0.76; *M* = 3.52, SD = 0.71).

An established instrument was used to measure personal norms (van der Werff et al., 2013a,b). In Study 1 personal norms to engage in each behavior were measured with three items. Participants indicated on a five-point scale, from *1 – totally disagree* to *5 – totally agree*, to what extent they feel obliged to recycle (e.g., “I feel morally obliged to recycle waste”; α = 0.69, *M* = 3.39, SD = 0.84), use sustainable transportation (e.g., “I feel morally obliged to choose environmentally friendly transportation means”; α = 0.76, *M* = 3.33, SD = 0.89), and purchase sustainably produced goods (e.g., “I feel morally obliged to choose products that are produced in the least environmentally harmful way”; α = 0.73, *M* = 3.31, SD = 0.86). In Study 2, due to the limited length of the questionnaire, we measured personal norms to engage in each behavior with single items, using the same five-point scale as in Study 1 (adapted from van der Werff et al., 2013a). Using single items is a valid way to measure personal norms (Schwartz, 1977). We tested to what extent participants feel obliged to recycle non-refundable plastics (“I feel morally obliged to recycle non-refundable plastics”; *M* = 3.13, SD = 1.21), cycle to school (“I feel morally obliged to cycle to school”; *M* = 2.26, SD = 1.14) and purchase organic food products (“I feel morally obliged to purchase organic food products”; *M* = 2.70, SD = 0.98). In Study 3, as in Study 1, we used three items to measure personal norms to engage in each behavior. Participants indicated to what extent they feel obliged to not drink bottled water (e.g., “I feel morally obliged to not drink bottled water”; α = 0.77, *M* = 2.96, SD = 0.99). In Study 1 and Study 3 we took the mean of these items to calculate personal norms; higher values indicate stronger personal norms to engage in each behavior.

Participants indicated on a five-point scale, from *1 – never or almost never* to *5 – always or almost always*, how often during the past two months they recycled waste (*M* = 3.02, SD = 1.19), chose environmentally friendly transportation means (*M* = 3.12, SD = 1.14) and chose products that are produced in the least environmentally harmful way (*M* = 2.98, SD = 1.11) in Study 1; how often during the period of the past four weeks they recycled non-refundable plastics (*M* = 3.00, SD = 1.42), cycled to school (*M* = 1.67, SD = 1.15) and purchased organic food products (*M* = 2.68, SD = 1.01) in Study 2; and how often during the period of the past four weeks they drank water from the tap or well (*M* = 4.40, SD = 0.89) in Study 3; higher values indicate stronger engagement in each behavior.

Confirmatory factor analysis indicated that in all three studies the items measuring biospheric values and environmental self-identity, also personal norms in Study 1 and Study 3, correlated stronger with their respective scales than with the other scales, after controlling for self-correlations (Supplementary Material 3). This suggests that biospheric values, environmental self-identity and personal norms discriminate well from each other. We did not test the discriminant validity of personal norms in Study 2, because each personal norm was measured with single item.

No reversed coded items compose above indicated instruments. The full item list can be found in Open Science Foundation repository: https://osf.io/yxfjz/?view_only=5bf95276c67a4984a8fb76cfe201abb7.

### Ethics Statement

All studies presented in the paper were conducted in accordance with the recommendations of and approved by the ethics committee at the Mykolas Romeris University [protocol number: 3A(11.21-32002)-129)]. Procedures applied in this research comply with the national and international research ethics standards (i.e., Regulations of Psychological Testing in Lithuania; American Psychological Association Ethics Code; Helsinki declaration). An informed consent (in written or electronic form) was obtained from study participants’ parents or legal guardians. Before starting to fill in the online questionnaires, participants were informed that they are not obliged to participate even if their parents gave consent for participation. Moreover, participants were informed that they can freely opt out from the study at any stage. Participants were briefly informed about the aims of the research, namely, to explore the attitudes of young people toward pro-environmental behavior. Participants were informed that their data will be kept confidentially and as soon as all the necessary stages of data processing are completed, the personal information (participants’ names in Study 1 and special ID codes in Study 2 and 3) will be permanently removed with no possibility to restore personal information. The final datasets do not allow to track the identity of the participants and are therefore anonymous.

### Analytic Strategy

We used the 23rd version of SPSS to calculate descriptive statistics and correlations, and to perform confirmatory factor analyses. To investigate the discriminant validity of the theoretical constructs, namely biospheric values, environmental self-identity and personal norms to act pro-environmentally, we used a confirmatory factor analysis, specifically the Oblique Multiple Group method (OMG; Nunnally, 1978). The OMG is commonly used to test the discriminant validity of above mentioned variables (Steg et al., 2012; van der Werff et al., 2013b, 2014b) and tests whether the data supports the *a priori* assignment of the items to the respective subscales/dimensions (Stuive et al., 2008).

To investigate the relationships between the key variables and pro-environmental behavior, we applied Structural Equation Modeling (SEM) in Mplus 8.2. (Muthén and Muthén, 2015). We tested whether biospheric values are related to various pro-environmental behaviors via environmental self-identity and personal norms. We applied the robust unweighted least squares estimator (ULSMV) together with the theta parameterization (theta parametrization is usually used when the model contains at least one ordinal variable). In the current research, ordinal variables were pro-environmental behaviors in all three studies and personal norms in Study 2, which are most appropriate procedures for such type of analysis (Muthén, 1993; Muthén and Asparouhov, 2002). The fit of the proposed model was evaluated with the following indices: RMSEA (the Root Mean Square Error of Approximation), CFI (the Comparative Fit Index), TLI (the Tucker-Lewis Index) and chi square (χ^2^). The fit of the model is considered acceptable when RMSEA ≤ 0.06, CFI ≥ 0.90, TLI ≥ 0.90, and when the chi square (χ^2^) value is not significant (Little, 2013). Noteworthy, the chi square indice may result in rejecting acceptable models because the indice is sensitive to sample peculiarities. It is therefore most important to consider CFI and TLI values, which are the derivatives of chi square when controlling for sample size (Hooper et al., 2008). We used a common way to report significance levels of SEM, namely with confidence intervals (Schreiber et al., 2006).

### Power Analysis

We employed *a priori* power analysis (Soper, 2019) to calculate the required sample size for the three studies. The analysis revealed that in order to conduct valid SEM analysis, when the effect size is.30, statistical power is.80 and the level of significance is.01, for Study 1 and Study 3 the recommended sample size is *n* = 161 (3 latent variables and 10 observed variables), and for Study 2, *n* = 133 (2 latent variables and 8 observed variables). The sample sizes of all studies exceeded the recommended threshold.

### Questionnaire Order Effects

In cross-sectional questionnaire studies it is important to control for possible order effects (Podasakoff et al., 2003). We used three different datasets across the three studies and applied relevant procedures to address this issue. In Study 1 and Study 2, the items were randomized within each scale measuring the key constructs (i.e., values, environmental self-identity, personal norms and pro-environmental behavior). In Study 3, two steps of randomization were followed. First, the items were randomized within each scale and second, the order of the scales measuring the key constructs was also randomized. In all three studies the demographic questions were presented at the beginning of the survey.

## Results

### Study 1

**FIGURE 3:**
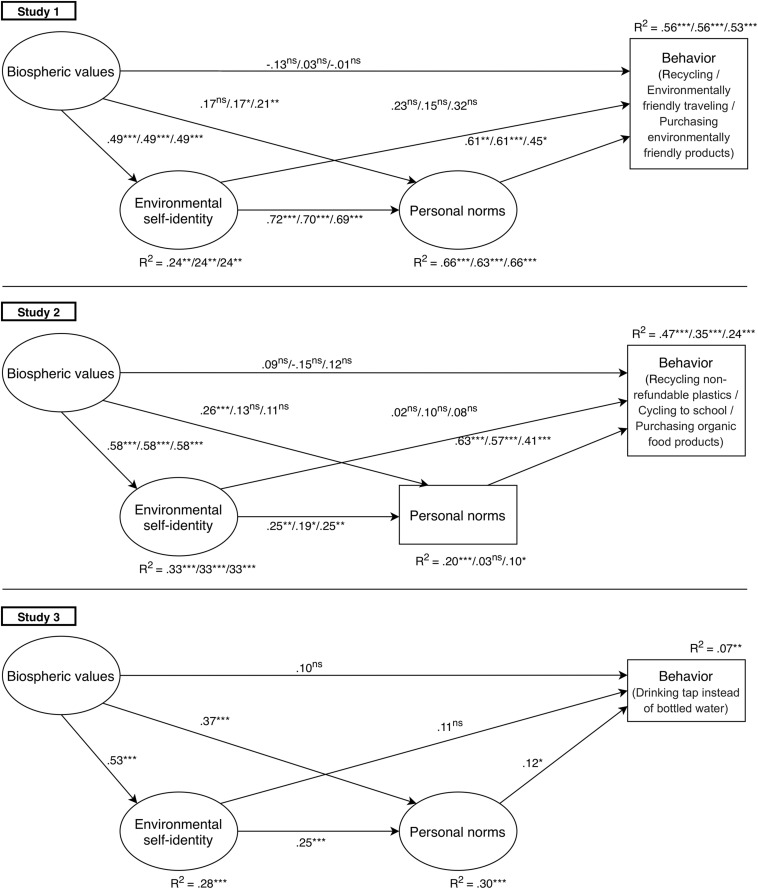
Standardized regression coefficients of the direct model paths for environmental considerations and pro-environmental behaviors in three studies in adolescents’ sample. Note. ns, non-significant; *p < 0.05, **p < 0.01, ***p < 0.001. In Study 1 and Study 2 each coefficient represents three behaviors, respectively.

Correlations between key variables are provided in Supplementary Material 2. The model of the relationships between biospheric values, environmental self-identity, personal norms and the three types of pro-environmental behaviors fitted the data sufficiently well [recycling: χ^2^(df) = 55.85(39), *p* = 0.04, CFI/TLI = 0.96/0.95, RMSEA [90% CI] = 0.04 [0.01,0.06]; environmentally friendly traveling: χ^2^(df) = 57.13(39), *p* = 0.03, CFI/TLI = 0.96/0.95, RMSEA [90% CI] = 0.04 [0.01,0.07]; and purchasing environmentally friendly products: χ^2^(df) = 64.19(39), *p* = 0.01, CFI/TLI = 0.95/0.92, RMSEA [90% CI] = 0.05 [0.03,0.07]; Figure 3].

Biospheric values were indirectly related to the three pro-environmental behaviors via environmental self-identity and personal norms (Table 2). Specifically, biospheric values explained 24% of the variance in environmental self-identity, which in turn was strongly related to personal norms. Together, biospheric values and environmental self-identity explained 66, 63, and 66% of the variance in personal norms to recycle, travel environmentally friendly and purchase sustainably produced goods, respectively. Personal norms, in turn, explained 56, 56, and 53% of variance in the three respective behaviors. In addition, biospheric values were also directly and moderately strongly related to the personal norms to travel environmentally friendly and to purchase environmentally friendly products.

**TABLE 2:** The indirect effects of adolescents’ biospheric values and environmental self-identity on pro-environmental behaviors.

**Study 1**			**Study 2**			**Study 3**		
	**→Behavior**			**→Behavior**			**→Behavior**	
**Indirect paths**	**Estimate [95% CI]**	**SE**	**Indirect paths**	**Estimate [95% CI]**	**SE**	**Indirect paths**	**Estimate [95% CI]**	**SE**

**Recycling**			**Recycling non-refundable plastic**			**Drinking water from the tap or well**		
(SUM)	**0.43 [0.29;0.62]**	0.08	(SUM)	**0.27 [0.18;0.38]**	0.05	(SUM)	**0.12 [0.05;0.19]**	0.03
BIO→ESI→ PN→	**0.22 [0.10;0.42]**	0.09	BIO→ESI→ PN→	**0.09 [0.03;0.15]**	0.03	BIO→ESI→ PN→	**0.02 [0.001;0.03]**	0.01
BIO→ESI→	0.11 [−0.09;0.30]	0.10	BIO→ESI→	0.01 [−0.06;0.11]	0.04	BIO→ESI→	0.06 [−0.002;0.12]	0.03
BIO→ PN→	0.10 [−0.01;0.26]	0.07	BIO→ PN→	**0.16 [0.07;0.26]**	0.05	BIO→ PN→	**0.04 [0.004;0.09]**	0.02
ESI→ PN→	**0.44 [0.21;0.81]**	0.16	ESI→ PN→	**0.16 [0.05;0.26]**	0.06	ESI→ PN→	**0.03 [0.002;0.06]**	0.02

**Environmentally friendly traveling**			**Cycling to school**					

(SUM)	**0.38 [0.26;0.53]**	0.07	(SUM)	0.05 [−0.09;0.18]	0.07			
BIO→ESI→ PN→	**0.21 [0.12;0.39]**	0.07	BIO→ESI→ PN→	**0.06 [0.01;0.13]**	0.03			
BIO→ESI→	0.08 [−0.08;0.19]	0.07	BIO→ESI→	0.06 [−0.07;0.18]	0.06			
BIO→ PN→	0.10 [−0.002;0.22]	0.06	BIO→ PN→	−0.07 [−0.16;0.01]	0.04			
ESI→ PN→	**0.42 [0.28;0.70]**	0.11	ESI→ PN→	**0.11 [0.01;0.21]**	0.05			

**Purchasing environmentally friendly products**			**Purchasing of organic food products**					

(SUM)	0.41 [0.29;0.56]	0.07	(SUM)	**0.15 [0.04;0.25]**	0.05			
BIO→ESI→ PN→	**0.15 [0.03;0.30]**	0.07	BIO→ESI→ PN→	**0.06 [0.02;0.10]**	0.02			
BIO→ESI→	0.16 [−0.01;0.38]	0.10	BIO→ESI→	0.05 [−0.05;0.14]	0.05			
BIO→ PN→	**0.10 [0.004;0.24]**	0.06	BIO→ PN→	0.04 [−0.01;0.11]	0.03			
ESI→ PN→	**0.31 [0.06;0.59]**	0.14	ESI→ PN→	**0.10 [0.03;0.17]**	0.04			

*CI, confidence interval; SE, standard error; BIO, biospheric values; ESI, environmental self-identity; PN, personal norm; SUM, cumulative indirect effect. Statistically significant values are marked in bold.*

Study 1 provides the first evidence that adolescents’ biospheric values can facilitate, via environmental self-identity and personal norms, general pro-environmental behaviors such as recycling, environmentally friendly traveling and purchasing environmentally friendly products. In Study 2, we test whether the results can be replicated for more specific pro-environmental behaviors. In addition, we targeted a different region in Lithuania (Figure 2) to cross-validate our findings.

### Study 2

All correlations between key variables are provided in Supplementary Material 2. The model of the relations between biospheric values, environmental self-identity, personal norms and three specific pro-environmental behaviors fitted the data sufficiently well [recycling non-refundable plastics χ^2^(df) = 48.53(23), *p* = 0.001, CFI/TLI = 0.93/0.90, RMSEA [90% CI] = 0.06 [0.03,0.08]; cycling to school χ^2^(df) = 35.71(23), *p* = 0.04, CFI/TLI = 0.97/0.95, RMSEA [90% CI] = 0.04 [0.01,0.06]; and purchasing organic food products χ^2^(df) = 43.41(23), *p* = 0.01, CFI/TLI = 0.95/0.92, RMSEA [90% CI] = 0.05 [0.03,0.07]; Figure 3].

Biospheric values were indirectly related to the three pro-environmental behaviors via environmental self-identity and personal norms (Table 2). Specifically, biospheric values explained 33% of the variance in environmental self-identity, which in turn was related to personal norms with the strength varying from small to moderate. Together, biospheric values and environmental self-identity explained 20, 3, and 10% of the variance in personal norms to recycle non-refundable plastics, cycle to school and purchase organic food products, respectively. Personal norms were strongly related to the respective behaviors and explained, accordingly, 47%, 35% and 24% of variance in these behaviors. Also, biospheric values were directly and moderately strongly related to moral obligation to recycle non-refundable plastics.

In Study 2, we found that biospheric values can also explain, via environmental self-identity and personal norms, a significant amount of variance in adolescents’ more specific pro-environmental behaviors, namely recycling non-refundable plastic, cycling to school and purchasing organic food products. Yet, the relationship between environmental self-identity and the three types of personal norms was of small to moderate strength and weaker than in Study 1. In Study 3, we test whether the same relationships hold for another specific pro-environmental behavior, namely drinking water from the tap. Again, we target a different region in Lithuania (Figure 2) to cross-validate the findings.

### Study 3

Correlations between key variables are provided in Supplementary Material 2. The model of the relations between biospheric values, environmental self-identity, personal norms and a specific pro-environmental behavior – to drink tap water or water from the well - fitted the data sufficiently well [χ^2^(df) = 155.98(39), *p* < 0.001, CFI/TLI = 0.92/0.89, RMSEA [90% CI] = 0.06 [0.04,0.07]; Figure 3].

Biospheric values were indirectly related to behavior via environmental self-identity and personal norms (Table 2). Specifically, biospheric values were strongly related to and explained 28% of the variance in environmental self-identity, which in turn was moderately strongly related to personal norms. Next, biospheric values and environmental self-identity explained 30% of the variance in personal norms to not drink bottled water. Personal norms were rather weakly related to the drinking water from the tap or well behavior and explained 12% of the variance in this behavior. Also, biospheric values were moderately directly related to personal norms.

In Study 3 we again found that biospheric values could explain adolescents’ very specific pro-environmental behavior, namely drinking less bottled water, via environmental self-identity and personal norms.

## General Discussion

Biospheric values, environmental self-identity and personal norms can strengthen people’s intrinsic motivation to engage in various pro-environmental behaviors and could therefore be targeted in order to effectively promote pro-environmental behavior (Ruepert et al., 2016; Steg, 2016; van der Werff and Steg, 2016). It is important to study whether environmental considerations influence pro-environmental behaviors of adolescents too, since it is crucial to motivate the youth and future generations to act pro-environmentally to address global environmental crisis. We carried out three studies to find out. Overall, across three studies in different regions in Lithuania, we have found initial evidence that general environmental considerations can motivate adolescents to act pro-environmentally.

First, in all three studies we found that adolescent’s biospheric values can be distinguished from altruistic, egoistic and hedonic values. This extends previous evidence which showed that universalism values, which encompass both biospheric and altruistic values, can be empirically distinguished from other values in adolescents (Schwartz et al., 2001; Liem et al., 2010; Schwartz, 2012b; Cieciuch et al., 2013; Paez and De-Juanas, 2015). Our finding that biospheric values in particular can be distinguished from other values suggests that biospheric values already form a distinct value type in adolescence. Also, we found that adolescents’ biospheric values, environmental self-identity and personal norms to act pro-environmentally can be empirically distinguished from each other, which has so far been shown for adults only (van der Werff et al., 2013a,b; Ruepert et al., 2016; van der Werff and Steg, 2016). This indicates that these moral and identity structures, although still under development and unstable, can already be distinguished among adolescents. Next, most importantly, we found that adolescents’ biospheric values were associated to various pro-environmental behaviors via environmental self-identity and personal norms to act pro-environmentally.

We found that environmental considerations were associated with general (e.g., purchasing environmentally friendly products) as well as more specific (e.g., purchasing organic food products) environmental behaviors. This provides support that general environmental considerations, such as biospheric values and environmental self-identity, can explain a large variety of behaviors. Yet, we also found that more general behaviors, for example recycling (Study 1), were more strongly related to biospheric values, environmental self-identity and personal norms than more specific behaviors, for example recycling non-refundable plastics (Study 2) and drinking water from the tap (Study 3). Specifically, environmental self-identity was less strongly associated with personal norms to engage in these specific behaviors, especially cycling to school. Also, the relationship between personal norm to drink tap water and the respective behavior was relatively weak. The principle of compatibility implies that constructs are more strongly related if they are measured at the same level of generality or specificity (Ajzen, 1996). Yet, this could not explain why in some cases we found that general constructs, such as biospheric values, were strongly related to specific constructs, such as the personal norm to drink tap water (see also Ruepert et al., 2016). Rather, we propose that general environmental considerations determine a general tendency to act pro-environmentally, yet other factors play a role once people consider engaging in a specific behavior (Trope and Liberman, 2012; Rim et al., 2013). Even if people’s environmental considerations are strong, other factors may prevent them from acting upon these considerations. For example, pro-environmental behavior can be relatively costly (e.g., purchasing organic products) and adolescents may simply not have control over certain behaviors (e.g., choice for the means of transportation). Indeed, perceived behavioral control (i.e., thinking that one has means and resources to perform certain behavior) could be the factor that is important in explaining why adolescents do not adopt certain pro-environmental behaviors (Theory of Planned Behavior; Fishbein and Ajzen, 1975; Ajzen and Fishbein, 1980, 2005). Perceived risks to behavior such as safety (e.g., to drink tap water instead of bottled; van der Linden, 2013) could also explain why adolescents (not)engage in these behaviors. Furthermore, social norms, especially among peers, could influence whether or not adolescents engage in specific behaviors. For example, they may be reluctant to drink tap water if social norms to drink bottled water prevails among adolescents. Relatedly, environmental considerations may better predict behaviors that are less constrained by situational barriers and/or done mostly in private. Thus, private behaviors, for example recycling or taking shorter showers, may sometimes be more easily adopted and performed. Future studies could examine which factors and to what extent moderate the relationship between biospheric values and various pro-environmental behaviors of adolescents.

Interestingly, we found that in all three studies adolescents’ biospheric values were stronger than their egoistic values and, in some cases, than their hedonic values (Study 3), while previously research has suggested that adolescents’ biospheric values may be surpassed by their self-enhancement values (Schwartz, 2012b; Vecchione et al., 2019). Our findings in fact indicate that adolescents have rather strong biospheric values. At the same time, a comparison with a recent study in Lithuania with adults (Balundë et al., 2019; Supplementary Material 4) reveals that biospheric and altruistic values of adolescents in the current study were slightly weaker and egoistic values were slightly stronger compared to adults; for hedonic values there was no such consistent pattern. These results are in line with previous evidence that biospheric values may be weaker in adolescence than later in life (Schwartz, 2012b; Vecchione et al., 2019). Yet, we cannot test across the two studies whether there are significant differences between the values of adolescents and adults. Overall, our findings suggest that adolescents may already have relatively strong biospheric values, but these values could still become stronger later in life. Future studies could test whether biospheric as well as other values, environmental self-identity, personal norms and pro-environmental behavior significantly differ between adolescents and adults. Also, longitudinal studies are needed to examine how these key constructs change throughout the lifetime of individuals and across different cohorts and which key factors influence these changes. For example, increasing societal debate on climate issues could potentially strengthen the environmental considerations and/or the effects of these considerations on environmental behavior.

The current findings have important implications for policies to promote adolescents’ pro-environmental behavior. Specifically, we show that biospheric values could be a gateway for adolescents’ many pro-environmental behaviors. Biospheric values, environmental-identity, and personal norms to act pro-environmentally are still forming in adolescence, and it could be the best time to strengthen them. Several directions for policy can be distinguished. First, there is preliminary evidence that education about nature makes adolescents feel more connected to nature (e.g., Liefländer et al., 2013; Jordan and Chawla, 2019) and could potentially strengthen their biospheric values (Martin and Czellar, 2017). Thus, policies could aim at strengthening adolescents’ biospheric values via environmental education (Liefländer et al., 2013) and activities in nature (Collado et al., 2013). This could be relevant for higher and university education too. There are already programs appearing in higher formal education such as environmental psychology, sustainable leadership, and sustainable development, among others, that target sustainability issues and potentially can strengthen environmental considerations of young people. Future studies could look at the effects of such programs on the students’ biospheric values, environmental self-identity and personal norms. Second, environmental self-identity could potentially be strengthened by reminding people of their past pro-environmental behavior, even if behaviors were rather rarely performed (van der Werff et al., 2014a,b; Lacasse, 2016; Fanghella et al., 2019). This could be done, for example, by evaluating adolescents’ pro-environmental behaviors with self-reports (e.g., in school settings), that they most certainly would engage in, and then providing them with feedback about their pro-environmental behavior. Also, some unique behaviors (e.g., participating in climate march or volunteering for forest clean-up project) that could strongly signal adolescents’ environmental self-identity could be included in the evaluation too. Third, perceived peers’ social norms to act pro-environmentally could enhance adolescents’ personal norms and their pro-environmental behavior (Collado et al., 2017), especially because adolescents are very susceptive to peer influence (Kerr et al., 2003). An effective measure may be to give adolescents feedback about pro-environmental behavior of their peers. This could be done similarly as in studies with adults where participants of a recycling program were getting feedback about the recycling behavior of their neighbors; knowing that others recycled more increased people’s recycling behavior (Schultz, 1999). Future studies could test whether such different interventions are effective to strengthen adolescents’ biospheric values and environmental self-identity and in turn whether there is an effect of such interventions on pro-environmental behavior. Fourth, besides strengthening environmental considerations, we suggest that it is important to address contextual factors such as removing barriers for specific pro-environmental behaviors (e.g., ensuring easy access to tap water). Yet future studies are needed to test whether environmental education and removing barriers for certain behaviors can indeed lead to more pro-environmental behavior.

Some limitations should be considered when interpreting the current findings. First, we cannot draw definite conclusions about causal relationships between biospheric values, environmental self-identity, personal norms and environmental behaviors. Past studies give initial evidence of causal relationships between these variables. For example, universalism values (encompassing biospheric and altruistic values) measured in one time point predicted environmental behavior of adults measured after a year (Thøgersen and Ölander, 2002). Also, manipulating environmental self-identity in experiments resulted in changes in environmental behavior (van der Werff et al., 2014b). Future studies could test whether these results can be replicated for adolescents. Second, the current research was conducted with adolescents in Lithuania; future studies could test whether this relationship holds for adolescents in other parts of the world. Third, we did not measure how adolescents perceived the studied behaviors, for example as easy or difficult. Perceived difficulty of behaviors could potentially explain why biospheric values, environmental self-identity and personal norms are related stronger to some behaviors than others. Also, the extent to which adolescents think that certain behaviors are common among their peers could influence the relationships between their biospheric values and these behaviors. Fourth, we studied how important people find protecting nature and the environment in general, without distinguish for which reasons. Follow-up research could test whether, for example, adolescents engage in different types of pro-environmental behavior depending on the reasons why they value the environment, including for selfish reasons (i.e., anthropocentric) or for the wellbeing of nature itself (i.e., ecocentric; Thompson and Barton, 1994). For example, future studies could test which motivation, ecocentric, anthropocentric or both drives young people to recycle, chose environmentally friendly transportation means, among others. Fifth, despite the efforts to reduce chances of deceitful answers and the effect of social desirability these biases could potentially affect the results of the studies. Although, according to recent study the effect of social desirability bias in pro-environmental behavior studies is rather weak (Vesely and Klöckner, 2020), it is nevertheless important to consider this possible limitation. Future studies could test whether this potential compound affects the relationship between biospheric values, environmental self-identity, personal norms and pro-environmental behavior in adolescents’ studies. This is possible through controlling tested models/relationships for general social desirability as well as testing to what extent social norms moderate the relationships between social desirability and variables in question (Vesely and Klöckner, 2020). Sixth, in this study we targeted environmental sustainability in particular, but it is also important to study which factors motivate people to engage in other types of sustainable actions (e.g., reducing inequalities) on an individual as well as organizational and institutional level (e.g., sustainable businesses and industry). Future studies could test to what extent different values, namely, biospheric, altruistic, egoistic and hedonic, on an individual as well as a group level, explain different aspects of sustainability (i.e., environmental, social, and economic; United Nations, 1987; United Nations General Assembly [UNGA], 2012) or sustainable development goals (e.g., ensure sustainable consumption, reduce inequality, promote sustainable economic growth, etc.; United Nations, 2015). For example, altruistic values could be particularly important for explaining protection of marginalized groups.

## Conclusion

We provide evidence that adolescents’ environmental behavior can be rooted in their biospheric values, environmental self-identity and personal norms to act pro-environmentally. The findings suggest that policies aimed at promoting pro-environmental behavior of adolescents may benefit from targeting biospheric values, environmental self-identity and personal norms, thereby strengthening adolescents’ intrinsic motivation to act pro-environmentally. Accordingly, we proposed some future directions for such policies. Also, this study extends previous research on biospheric values, environmental self-identity and personal norms beyond adult samples.

## Data Availability Statement

The datasets generated in this study can be found in online repositories. The names of the repository/repositories and accession number(s) can be found below: Open Science Foundation repository, https://osf.io/yxfjz/?view_only=5bf95276c67a4984a8fb76cfe201abb7.

## Ethics Statement

The studies involving human participants were reviewed and approved by The Ethics Committee at the Mykolas Romeris University [protocol number: 3A(11.21-32002)-129)]. Written informed consent to participate in this study was provided by the participants’ legal guardian/next of kin.

## Author Contributions

AB and IT-K performed the material preparation, data collection, and analysis. AB wrote the first draft of the manuscript. GP consulted regarding the concept of the manuscript and made multiple critical revisions to its versions. All authors commented on previous versions of the manuscript and read and approved the final manuscript.

## Conflict of Interest

The authors declare that the research was conducted in the absence of any commercial or financial relationships that could be construed as a potential conflict of interest.

## Footnotes

1 For other values, a few items correlated slightly stronger with other value scales (see Supplementary Material 1).
