# Supplementary material for: Sustainability in Youth: Environmental Considerations in Adolescence and Their Relationship to Pro-environmental Behavior
Source: Front Psychol. 2020 Nov 2;11:582920. doi: 10.3389/fpsyg.2020.582920 (PMC7667260; doi:10.3389/fpsyg.2020.582920)
Supplement: Supplementary file 1 [file Table_1.DOCX]

Supplementary Material 1

# Supplementary Table 1.

*Correlations between the value items and the corresponding value scales.*

|  | Study 1 | | | |  | Study 2 | | | |  | Study 3 | | | |
| --- | --- | --- | --- | --- | --- | --- | --- | --- | --- | --- | --- | --- | --- | --- |
| Scale | BIO | ALT | EGO | HED |  | BIO | ALT | EGO | HED |  | BIO | ALT | EGO | HED |
| Biospheric values |  |  |  |  |  |  |  |  |  |  |  |  |  |  |
| Respecting the earth | **.622** | .515 | .339 | .366 |  | **.629** | .525 | .148 | .245 |  | **.638** | .561 | .189 | .284 |
| Unity with nature | **.664** | .486 | .366 | .364 |  | **.606** | .473 | .172 | .214 |  | **.642** | .552 | .215 | .299 |
| Protecting the environment | **.694** | .533 | .400 | .411 |  | **.660** | .529 | .193 | .260 |  | **.668** | .522 | .166 | .268 |
| Preventing pollution | **.661** | .507 | .377 | .387 |  | **.619** | .487 | .190 | .212 |  | **.647** | .535 | .167 | .299 |
| Altruistic values |  |  |  |  |  |  |  |  |  |  |  |  |  |  |
| Equality | .490 | **.531** | .275 | .460 |  | .409 | **.490** | .176 | .235 |  | .476 | **.501** | .153 | .289 |
| A world at peace | .505 | **.510** | .314 | .424 |  | .501 | **.504** | .163 | .243 |  | **.558** | .499 | .169 | .358 |
| Social justice | **.608** | .537 | .335 | .398 |  | .553 | **.561** | .204 | .247 |  | **.559** | .539 | .219 | .299 |
| Helpful | .438 | **.479** | .282 | .323 |  | **.551** | .543 | .179 | .239 |  | **.576** | .514 | .189 | .299 |
| Egoistic values |  |  |  |  |  |  |  |  |  |  |  |  |  |  |
| Social power | 309 | .228 | **.493** | .293 |  | .114 | .098 | **.391** | .266 |  | .041 | .023 | **.335** | .199 |
| Wealth | .277 | .195 | .404 | **.410** |  | .019 | .020 | .284 | **.379** |  | .071 | .088 | .279 | **.358** |
| Authority | .362 | .273 | **.496** | .367 |  | .101 | .100 | **.349** | .291 |  | .089 | .094 | **.362** | .238 |
| Influential | .358 | .292 | **.442** | .306 |  | .264 | .305 | **.339** | .318 |  | .247 | .226 | **.330** | .283 |
| Ambitious | **.545** | .519 | .417 | .529 |  | **.381** | .379 | .173 | .287 |  | .474 | **.480** | .145 | .291 |
| Hedonic values |  |  |  |  |  |  |  |  |  |  |  |  |  |  |
| Pleasure | .472 | .456 | .398 | **.696** |  | .233 | .286 | .326 | **.565** |  | .308 | .335 | .275 | **.538** |
| Enjoying life | 330 | .377 | .365 | **.608** |  | .306 | .296 | .271 | **.532** |  | .371 | .412 | .252 | **.500** |
| Self-indulgent | .344 | .370 | .379 | **.665** |  | .159 | .140 | .328 | **.511** |  | .184 | .188 | .294 | **.474** |

*Note*. Correlation coefficients are corrected for self-correlation and test-length. The highest correlation coefficients of each item are marked in bold. The tested item grouping explains 68.43% of variance in Study 1, 63.44% in Study 2 and 63.61% in Study 3. BIO – biospheric values; ALT – altruistic values; EGO – egoistic values; HED – hedonic values.

In Study 1, one item measuring altruistic values, namely “Social justice”, correlated more strongly with the biospheric values scale than with the altruistic values scale. This relationship is not striking since biospheric and altruistic values belong to the same self-transcendence values cluster (Schwartz, 1992). One item measuring egoistic values, namely “Ambitious”, correlated stronger with the biospheric values scale than the egoistic values scale. Similarly, studies with adult samples in post-socialist countries, including Lithuania, Czechia and Hungary, have found that this item correlated stronger with the altruistic values scale (de Groot and Steg, 2007; de Groot et al., 2012; Balundė et al., 2019). In Study 2, one item measuring altruistic values, namely “Helpful”, correlated more strongly with the biospheric values scale than with the altruistic values scale, and the item “Ambitious”, which is intended to measure egoistic values, correlated more strongly with the biospheric values scale than with the egoistic values scale (see above for possible explanations). In Study 3, the items measuring altruistic values “Social justice” and “Helpful” correlated more strongly with the biospheric values scale. These findings, again, can be explained by the fact that both type of values are self-transcendence values. Also, in this study “A world at peace” altruistic values item correlated stronger with the biospheric values scale. These results were in line with the findings in adults’ sample in Lithuania, Czechia and Hungary, (de Groot and Steg, 2007; de Groot et al., 2012; Balundė et al., 2019). Yet the question is whether the discrepancies from the theory regarding altruistic values items are specific to the sample of young people particularly in Lithuania. Future studies are needed to test whether this can be replicated elsewhere. Since in all our three studies biospheric values formed reliable scale we kept the scale of biospheric values as it is intended based on theoretical grounds and prior research.
