# Supplementary material for: Sustainability in Youth: Environmental Considerations in Adolescence and Their Relationship to Pro-environmental Behavior
Source: Front Psychol. 2020 Nov 2;11:582920. doi: 10.3389/fpsyg.2020.582920 (PMC7667260; doi:10.3389/fpsyg.2020.582920)
Supplement: Supplementary file 2 [file Table_2.DOCX]

Supplementary Material 2

# Supplementary Table 2.

*Bivariate correlations between biospheric values, environmental self-identity, personal norms and pro-environmental behaviors.*

| Study 1 | | | | | | | | |
| --- | --- | --- | --- | --- | --- | --- | --- | --- |
|  | 1. | 2. | 3. | 4. | 5. | 6. | 7. | 8. |
| 1. Biospheric values | - |  |  |  |  |  |  |  |
| 2. Environmental self-identity | .40** | - |  |  |  |  |  |  |
| 3. Personal norm (recycling) | .41** | .59** | - |  |  |  |  |  |
| 4. Personal norm (travel) | .42** | .61** | .80** | - |  |  |  |  |
| 5. Personal norm (consumption) | .44** | .59** | .78** | .79** | - |  |  |  |
| 6. Behavior (recycling) | .26** | .55** | .57** | .54** | .55** | - |  |  |
| 7. Behavior (travel) | .37** | .54** | .50** | .63** | .61** | .41** | - |  |
| 8. Behavior (consumption) | .35** | .57** | .54** | .55** | .57** | .53** | .57** | - |
| γ1 | -.53 | -.17 | -.37 | -.33 | -.21 | .03 | -.03 | -.01 |
| γ2 | -.47 | -.13 | .29 | .02 | .05 | -.98 | -.83 | -.72 |
| Study 2 | | | | | | | | |
|  | 1. | 2. | 3. | 4. | 5. | 6. | 7. | 8. |
| 1. Biospheric values | - |  |  |  |  |  |  |  |
| 2. Environmental self-identity | .44** | - |  |  |  |  |  |  |
| 3. Personal norm (recycling) | .36** | .31** | - |  |  |  |  |  |
| 4. Personal norm (cycling) | -.02 | .09 | .12* | - |  |  |  |  |
| 5. Personal norm (organic food products) | .23** | .23** | .33** | .17** | - |  |  |  |
| 6. Behavior (recycling) | .31** | .27** | .60** | -.01 | .18** | - |  |  |
| 7. Behavior (cycling) | -.05 | .05 | -.03 | .49** | .04 | .07 | - |  |
| 8. Behavior (organic food products) | .23** | .21** | .14** | .04 | .41** | .29** | .05 | - |
| γ1 | -.43 | -.01 | -.22 | .56 | .04 | -.05 | 1.72 | -.04 |
| γ2 | -.69 | .09 | -.77 | -.53 | -.22 | -1.30 | 1.88 | -.58 |
| Study 3 | | | | | | | | |
|  | 1. | 2. | 3. | 4. |  |  |  |  |
| 1. Biospheric values | - |  |  |  |  |  |  |  |
| 2. Environmental self-identity | .43** | - |  |  |  |  |  |  |
| 3. Personal norm (to refuse drink bottled water) | .42** | .33** | - |  |  |  |  |  |
| 4. Behavior (drink tap water) | .17** | .14** | .15** | - |  |  |  |  |
| γ1 | -.91 | -.21 | -.05 | -1.62 |  |  |  |  |
| γ2 | .21 | .54 | -.43 | 2.39 |  |  |  |  |

*Note*. *p < .05, **p < .01; γ_1_ = skewness, γ_2_ = kurtosis.
