# Supplementary material for: Sustainability in Youth: Environmental Considerations in Adolescence and Their Relationship to Pro-environmental Behavior
Source: Front Psychol. 2020 Nov 2;11:582920. doi: 10.3389/fpsyg.2020.582920 (PMC7667260; doi:10.3389/fpsyg.2020.582920)
Supplement: Supplementary file 3 [file Table_3.DOCX]

Supplementary Material 3

# Supplementary Table 3.

*Correlations between the biospheric values, environmental self-identity and personal norms items.*

|  | Study 1 | | |  | Study 2 | |  | Study 3 | | |
| --- | --- | --- | --- | --- | --- | --- | --- | --- | --- | --- |
| Scale | BIO | ESI | PN | Scale | BIO | ESI | Scale | BIO | ESI | PN |
| Biospheric values |  |  |  | Biospheric values |  |  | Biospheric values |  |  |  |
| Respecting the earth | **.622** | .238 | .235 | Respecting the earth | **.629** | .288 | Respecting the earth | **.638** | .301 | .282 |
| Unity with nature | **.664** | .254 | .278 | Unity with nature | **.606** | .281 | Unity with nature | **.642** | .288 | .285 |
| Protecting the environment | **.694** | .343 | .310 | Protecting the environment | **.660** | .329 | Protecting the environment | **.668** | .314 | .294 |
| Preventing pollution | **.661** | .332 | .289 | Preventing pollution | **.619** | .301 | Preventing pollution | **.647** | .296 | .323 |
| Environmental self-identity |  |  |  | Environmental self-identity |  |  | Environmental self-identity |  |  |  |
| Item 1 | .306 | **.524** | .447 | Item 1 | .425 | **.437** | Item 1 | .402 | **.493** | .320 |
| Item 2 | .257 | **.540** | .334 | Item 2 | .181 | **.465** | Item 2 | .198 | **.501** | .115 |
| Item 3 | .312 | **.553** | .372 | Item 3 | .293 | **.518** | Item 3 | .300 | **.557** | .228 |
| Personal norms |  |  |  |  |  |  | Personal norms |  |  |  |
| Item 1 (recycling) | .311 | .456 | **.509** |  |  |  | Item 1 (refrain from bottled water use) | .306 | .247 | **.544** |
| Item 2 (recycling) | .298 | .413 | **.504** |  |  |  | Item 2 (refrain from bottled water use) | .295 | .225 | **.562** |
| Item 3 (recycling) | .224 | .284 | **.438** |  |  |  | Item 3 (refrain from bottled water use) | .287 | .190 | **.483** |
| Item 1 (travel) | .284 | .453 | **.543** |  |  |  |  |  |  |  |
| Item 2 (travel) | .369 | .450 | **.550** |  |  |  |  |  |  |  |
| Item 3 (travel) | .236 | .347 | **.463** |  |  |  |  |  |  |  |
| Item 1 (consumption) | .326 | .393 | **.527** |  |  |  |  |  |  |  |
| Item 2 (consumption) | .323 | .484 | **.513** |  |  |  |  |  |  |  |
| Item 3 (consumption) | .275 | .314 | **.462** |  |  |  |  |  |  |  |

*Note.* Correlation coefficients are corrected for self-correlation and test-length. The highest correlation coefficients of each item are marked in bold. BIO – biospheric values; ESI – environmental self-identity; PN – personal norms.
