# Supplementary material for: Sustainability in Youth: Environmental Considerations in Adolescence and Their Relationship to Pro-environmental Behavior
Source: Front Psychol. 2020 Nov 2;11:582920. doi: 10.3389/fpsyg.2020.582920 (PMC7667260; doi:10.3389/fpsyg.2020.582920)
Supplement: Supplementary file 4 [file Table_4.DOCX]

Supplementary Material 4

# Supplementary Table 4.

*Means of biospheric, altruistic, egoistic and hedonic values, and environmental self-identity among adults (Balundė et al., 2019) and adolescents (current research) in Lithuania*. *Data for these calculations are publicly available on Open Science Foundation repository. Link to the data is available from the abovementioned publication.*

|  | Biospheric values (M) | Altruistic values (M) | Egoistic values (M) | Hedonic values (M) | Environmental self-identity (M) |
| --- | --- | --- | --- | --- | --- |
| **AD** | **5.58** | **5.60** | **3.13** | **5.05** | ***5.25**  **** 3.84** |
| ADOL |  |  |  |  |  |
| Study 1 | 4.42 | 4.91 | 4.12 | 5.31 | 3.33 |
| Study 2 | 4.35 | 5.00 | 3.25 | 4.59 | 3.60 |
| Study 3 | 5.12 | 5.44 | 3.38 | 4.64 | 3.52 |
| **M(Study1, Study2, Study 3)** | **4.63** | **5.12** | **3.58** | **4.85** | **3.48** |
|  | AD>ADOL | AD>ADOL | AD<ADOL | AD>ADOL | AD>ADOL |

*Note*. *original value from 7-point scale; **7-point scale value converted to 5-point scale value. AD – adults; ADOL – adolescents.
